# Incidental intracranial meningiomas: a systematic review and meta-analysis of prognostic factors and outcomes

**DOI:** 10.1007/s11060-019-03104-3

**Published:** 2019-01-17

**Authors:** Abdurrahman I. Islim, Midhun Mohan, Richard D. C. Moon, Nisaharan Srikandarajah, Samantha J. Mills, Andrew R. Brodbelt, Michael D. Jenkinson

**Affiliations:** 1 Institute of Translational Medicine, University of Liverpool, Liverpool, UK; 2 Faculty of Health and Life Sciences, University of Liverpool, Liverpool, UK; 3 Department of Neurosurgery, The Walton Centre NHS Foundation Trust, Liverpool, UK; 4 Department of Neuroradiology, The Walton Centre NHS Foundation Trust, Liverpool, UK

**Keywords:** Asymptomatic, Incidental, Meningioma, Meta-analysis, Systematic review

## Abstract

**Background:**

Incidental discovery accounts for 30% of newly-diagnosed intracranial meningiomas. There is no consensus on their optimal management. This review aimed to evaluate the outcomes of different management strategies for these tumors.

**Methods:**

Using established systematic review methods, six databases were scanned up to September 2017. Pooled event proportions were estimated using a random effects model. Meta-regression of prognostic factors was performed using individual patient data.

**Results:**

Twenty studies (2130 patients) were included. Initial management strategies at diagnosis were: surgery (27.3%), stereotactic radiosurgery (22.0%) and active monitoring (50.7%) with a weighted mean follow-up of 49.5 months (SD = 29.3). The definition of meningioma growth and monitoring regimens varied widely impeding relevant meta-analysis. The pooled risk of symptom development in patients actively monitored was 8.1% (95% CI 2.7–16.1). Associated factors were peritumoral edema (OR 8.72 [95% CI 0.35–14.90]) and meningioma diameter ≥ 3 cm (OR 34.90 [95% CI 5.17–160.40]). The pooled proportion of intervention after a duration of active monitoring was 24.8% (95% CI 7.5–48.0). Weighted mean time-to-intervention was 24.8 months (SD = 18.2). The pooled risks of morbidity following surgery and radiosurgery, accounting for cross-over, were 11.8% (95% CI 3.7–23.5) and 32.0% (95% CI 10.6–70.5) respectively. The pooled proportion of operated meningioma being WHO grade I was 94.0% (95% CI 88.2–97.9).

**Conclusion:**

The management of incidental meningioma varies widely. Most patients who clinically or radiologically progressed did so within 5 years of diagnosis. Intervention at diagnosis may lead to unnecessary overtreatment. Prospective data is needed to develop a risk calculator to better inform management strategies.

**Electronic supplementary material:**

The online version of this article (10.1007/s11060-019-03104-3) contains supplementary material, which is available to authorized users.

## Introduction

With the widespread access and use of brain imaging, incidental asymptomatic meningiomas are fast becoming a modern medical issue for neurosurgeons and neuro-oncologists and patients are becoming the so-called Victims Of Modern Imaging Technology (VOMIT) [1, 2]. Their management consists of surgery, radiosurgery, fractionated radiotherapy and active monitoring. Recent consensus guidelines suggest active monitoring to be the most appropriate management strategy in the first instance; however, the frequency and duration of follow-up are not specified [3]. This leads to a variety of different monitoring strategies which has economic implications and is of uncertain patient benefit [4].

## Objective

To evaluate the outcomes of current management strategies of incidental intracranial meningiomas with particular emphasis on active monitoring and the timing of meningioma progression during follow-up.

## Methods

This systematic review was conducted in accordance with the Preferred Reporting Items for Systematic Reviews and Meta-analyses (PRISMA) statement [5], and has been registered with PROSPERO (CRD42017077928).

### Search strategy

A literature search, last updated 24/09/2017, was performed in Medline (Ovid), Embase (Ovid), the Cochrane Central Register of Controlled Trials, CINAHL Plus (EBSCO), the WHO International Clinical Trials Registry Platform and UK Clinical Trials Gateway. The search strategy utilized for Medline can be found in Online Resource 1. The strategy adopted for the other databases was altered appropriately and the term “meningioma” was used to search the study registries. The bibliographies of accepted records were examined for additional articles not identified in the initial search.

### Paper selection

The titles and abstracts of all results were screened independently by two authors (A.I.I. and M.M.) using the population, intervention, comparison, outcome and study design (PICOS) criteria outlined in Table 1. Where disagreements occurred, the senior author (M.D.J.) was consulted to adjudicate.

**Table 1:** PICOS inclusion criteria

Population	Patients ≥ 16 years of age diagnosed radiologically with an incidental asymptomatic intracranial meningioma/s. Neurofibromatosis type II associated and radiation-induced meningiomas were excluded					
Intervention	Active monitoring, surgery, SRS, *f*RT, or hospital discharge after the first inpatient/outpatient appointment					
Comparator	Not required					

Outcomes	Primary					Secondary
	Active monitoring	Surgery	SRS	*f*RT	Hospital discharge	All treatment options
	Progression and time to progression (clinical and radiological)	Treatment-related morbidity	Treatment-related morbidity	Treatment-related morbidity	Meningioma-related readmissions	Neurocognitive function
	Intervention and time to intervention	Meningioma grade	Recurrence	Recurrence		Quality of life
		Recurrence				
Study design	Randomized controlled trials and retrospective and prospective case series and cohort studies with ≥ 10 adult patients. Case reports were excluded					

*SRS* stereotactic radiosurgery, *f*RT fractionated radiotherapy

Relevant registered trials were categorized, based on recruitment status, into concluded or on-going. If concluded, disseminated results were examined. If yet to be disseminated, investigators were contacted for data involving incidental meningioma patients. On-going trials were excluded.

### Data extraction

A standardized pre-piloted proforma (Online Resource 2) was used to extract data from included studies. In the event that a study’s population was comprised in part of incidental meningioma patients, the corresponding author was contacted via email to request the raw study data. Data sets that subsequently remained incomplete were handled using the following imputation approach:

i. Studies in which incidental meningioma patients comprised ≥ 90% of the cohort, weighted averages were quoted and used for quantitative analysis
ii. Studies with a lower percentage were excluded

Data were inputted into Microsoft Excel v16.0 before being exported to statistical software for analysis.

### Data synthesis

Meningioma location was categorized into non-skull base and skull-base. Further subdivision according to the International Consortium on Meningioma classification system (Online Resource 3) was carried out where appropriate. Meningioma size was recorded as reported in each study. Volumetric measurements were converted to diametric measurements using the equation ${\text{mean}}~{\text{tumor}}~{\text{diameter}} = \sqrt[3]{{2~ \times {\text{volume}}}}$ and vice versa [6]. Diametric measurements were used for prognostication whereas changes in tumor size over time were performed using volumetry. Post-intervention morbidity and presenting symptoms were grouped into hierarchical domains where necessary. Extent of resection for patients who underwent surgery was categorized into gross total resection (GTR) (Simpson grades I–III) and subtotal resection (STR) (Simpson grades IV–V).

### Statistical analysis

Study-level data were collated and presented as number (percent), mean (standard deviation [SD]) or median (interquartile range [IQR]), based on the type of data. The pooled rates (95% confidence interval [95% CI]) of primary outcome measures were determined using the proportion meta-analysis function in StatsDirect v3.0, where proportions were transformed using the Freeman–Tukey double arcsine method [7], before an inverse-variance weighted random effects meta-analysis was performed. Heterogeneity across studies was estimated using the I^2^ statistic and classified into low (≤ 25%), moderate (~ 50%) and high (≥ 75%). Analysis of prognostic factors comprised three parts; firstly, the relationship between baseline covariates and outcomes were assessed on a non-weighted univariate level. Secondly, weighted meta-analyses were performed using risk difference (RD) as an effect size. Thirdly, the prognostic impact was assessed on a multivariate level using individual patient data (IPD).

#### Simple pooled analysis

Statistical differences for categorical variables were assessed using the Chi square test. Continuous variables were examined using the Mann–Whitney U test or Student’s *t* test. Differences were considered to be statistically significant at p < 0.05. These analyses were performed in an attempt to maintain data for single-arm prognostic or outcome cohorts with no relevant comparators. Data were analysed using SPSS v24.0.

#### Meta-analysis

The effect size measured was RD (95% CI) with forest plots used for graphical representation. If a 95% confidence interval included zero, analysis was deemed statistically non-significant. The Mantel–Haenszel model was used to combine the results from the studies included with weighting per study performed using random effects. Heterogeneity was measured and categorized as described above. All analyses were performed using RevMan v5.3.5.

#### Multivariable regression analysis

Pooling of IPD pertaining to ≥ 2 tested baseline variables was carried out wherever possible and statistical significance was assessed using a backward stepwise binary logistic regression analysis. Significance was set at p < 0.05. To account for heterogeneity across studies, a multi-level model was utilized with random effects at the level of studies. Odds ratios (OR) with 95% CI were calculated to determine effect size. Analyses were performed in Stata v14.1.

### Quality and bias assessment

Each study was assessed using the NIH Quality Assessment Tool for Observational Cohort and Cross-Sectional Studies [8], by two authors (A.I.I. and M.M.). This tool addresses internal validity criteria such as selection, measurement, and outcome biases. Results were compared and in case of discrepancies, the senior author (M.D.J.) was consulted. The strength of evidence for each primary outcome was subsequently examined using the Grading of Recommendations, Assessment, Development and Evaluations (GRADE) framework which consists of the following domains: (i) risk of bias, (ii) heterogeneity, (iii) directness, (iv) precision and (v) reporting bias. Heterogeneity was assessed using the I^2^ statistic, as described previously. Directness was evaluated using the PICO format. Examination of 95% CIs provided the primary approach to decisions regarding precision. For each treatment group, study-level reporting bias was assessed using Harbord’s and Begg’s tests and visualized for asymmetry using a funnel plot [9, 10]. Sensitivity analysis, using a trim-and-fill method [11], was utilized to recalculate pooled effect size if reporting bias was detected.

## Results

### Literature search and study characteristics

**Fig. 1:**
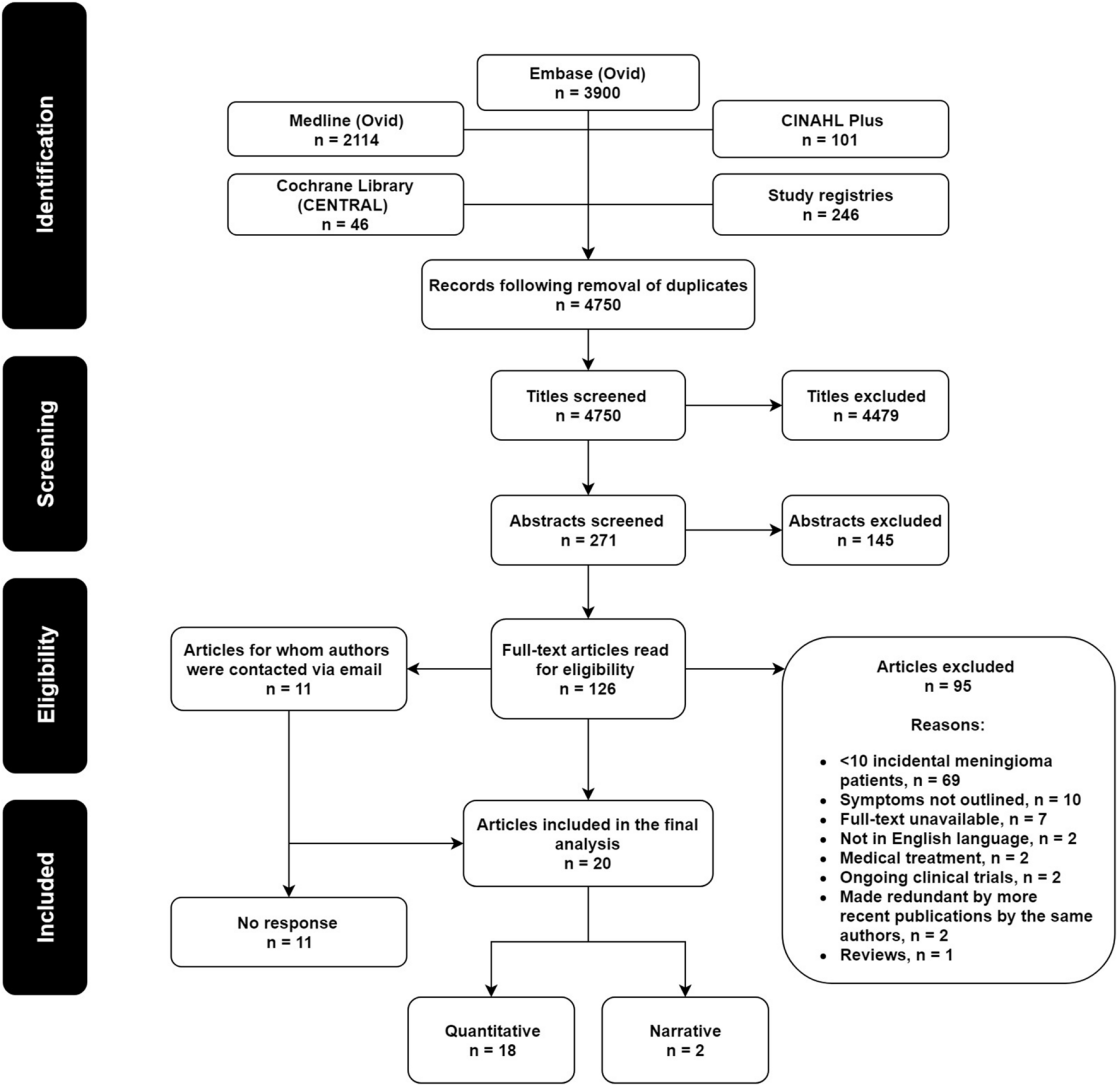
PRISMA flowchart demonstrating the study selection process

Figure 1 describes the study selection process. The initial number of studies included was 20. The corresponding authors for an additional 11 records were contacted. A duration of 3 months was allowed for responses and follow-up emails were sent to those expressing interest in providing data; however, no responses were received and therefore the final number of studies remained 20. The characteristics of these studies are summarized in Online Resource 4.

### Baseline characteristics

The overall number of incidental meningioma patients was 2130. Eighteen studies comprising 2061 (96.8%) patients were examined for quantitative analysis [12–29] and two studies (69 patients) were used for a narrative review of quality of life (QoL) and neurocognitive function (NCF) outcomes [30, 31]. The commonest indications for brain imaging amongst patients with available data (n = 745) were neurological deficit (14.4%), headache (13.6%), audiovestibular symptoms (11.8%) and head injury (10.3%). These were all deemed unrelated to the meningiomas discovered. Baseline characteristics are outlined in Table 2.

**Table 2:** Baseline clinical and radiological characteristics

No. of studies informing characteristic	No. of valid cases informing characteristic (%)	Characteristics		Total	Surgery	SRS	Active monitoring	P
18	2050	No. of patients (%)		2050	560 (27.3)	450 (22.0)	1040 (50.7)	
12	803 (39.2)	Mean age, years (SD)		63.1 (6.9)	61.5 (4.7)	54.9 (NR)^b^	64 (6.9)	< 0.001
17	1919 (93.6)	Sex, N (%)	Female	1526	294 (19.3)	375 (24.6)	857 (56.2)	< 0.001
			Male	393	164 (41.7)	75 (19.1)	154 (39.2)	
16	1465 (71.5)	Location, N (%)^a^	Non-skull base	1012	269 (26.6)	233 (23.0)	510 (50.4)	< 0.001
			Convexity	484	129	86	269	
			Parafalcine	247	55	71	121	
			Parasagittal	153	40	36	77	
			Tentorial	61	11	28	22	
			Intraventricular	24	3	12	9	
			Skull base	453	113 (24.9)	153 (33.8)	187 (41.3)	
			Anterior midline	113	30	43	40	
			Sphenoid wing	100	24	11	62	
			Posterior fossa—lateral and posterior	48	22	12	14	
			Posterior fossa—midline	143	18	87	34	
15	888 (43.3)	Mean diameter, cm (SD)		2.14 (0.61)	2.11 (0.42)	1.73 (NR)^b^	2.19 (0.66)	< 0.001
10	615 (30.0)	Calcification, N (%)	No	380	55 (14.5)	NR	325 (85.5)	0.774
			Yes	235	36 (15.3)	NR	199 (84.7)	
5	298 (14.5)	Tumor signal intensity, N (%)	Hyperintense	120	40 (33.3)	NR	80 (66.6)	0.237
			Iso/hypointense	178	48 (27.0)	NR	130 (73.0)	
12	1097 (53.5)	Peritumoral edema, N (%)	Yes	231	57 (24.7)	19 (8.2)	155 (67.1)	< 0.001
			No	866	135 (15.6)	370 (42.7)	361 (41.7)	

*NR* not reported, *SRS* stereotactic radiosurgery

^a^One study which dichotomized location into supratentorial and infratentorial was excluded [15]

^b^Available in one study which did not report SD [25]

### Treatment groups and outcomes

At initial presentation, three management strategies were identified; surgery (n = 560, 27.3%), stereotactic radiosurgery (SRS) (n = 450, 22.0%) and active monitoring (n = 1040, 50.7%). No patients received fractionated radiotherapy (*f*RT). The differences in characteristics amongst the three groups are summarized in Table 2.

#### Active monitoring

##### Follow-up regimens

Fifteen studies included patients who were actively monitored, of which only six described their follow-up regimens. A maximum follow-up duration for patients who did not experience radiological or clinical progression was not stated. Study-specific follow-up protocols are described in Table 3.

**Table 3:** Active monitoring protocols and terminology used to define growth during follow-up

Study	Protocol	Growth definition	
	Timing of scan following diagnosis	Measurement	Definition
Olivero et al. (1995) [13]	3 months → 9 months → 1–2 yearly	NR	NR
Go et al. (1998) [14]	NR	Diameter	≥ 0.5 cm
Niiro et al. (2000) [16]	NR	Diameter	≥ 0.5 cm
Yoneoka et al. (2000) [17]	NR	Volume	> 1 cm^3^/year
Nakamura et al. (2003) [18]	6 months → 1 yearly	NR	NR
Sonoda et al. (2004) [19]	3 months → 6 monthly	NR	NR
Hashiba et al. (2009) [22]	NR	Volume	> 15%
Jo et al. (2010) [23]	6 months → 1–2 yearly	Volume	> 25%
Jadid et al. (2014) [26]	1 yearly for a minimum of 10 years	Diameter	> 0.2 cm
Liu et al. (2015) [27]	3–12 monthly	NR	NR

*NR* not reported

##### Radiological and clinical progression

Follow-up times were reported for 683 patients, with a weighted mean of 49.5 months (SD = 29.3). During follow-up, 235/1040 (22.6%) meningiomas grew, according to each study-specific criterion for growth (Table 3). Time to first evidence of radiological progression was reported for 69 (29.4%) patients at a weighted mean of 28.5 months (SD = 7.5). Considering the heterogeneity of growth definitions, relevant meta-analyses were not performed.

For 432 patients, symptom status was not reported. Out of the remaining 608 patients, 66 patients developed symptoms (pooled proportion = 8.1% [95% CI 2.7–16.1], I^2^ = 88.6%). These included seizure (n = 8), motor deficit (n = 6), cognitive deficit (n = 3), visual deficit (n = 2) and cranial nerve palsy (n = 2). The nature of symptoms was not stated in the remaining 45. Differences in baseline characteristics amongst patient groups are shown in Online Resource 5. Forest plots can be found in Online Resource 6.

##### Intervention endpoints and timeframe for treatability

Intervention was recommended or carried out in 220 patients (pooled proportion = 24.8% [95% CI 7.5–48.0], I^2^ = 98.1%). Indications for treatment were radiological progression (n = 153), development of symptoms (n = 66) and patient preference (n = 1). Surgery was performed in 179 whilst SRS was the intervention of choice in the remainder (n = 30, 14.2%). Two patients were subject to surgery and adjuvant SRS. Weighted mean time-to-intervention, available for 175 patients, was 24.8 months (SD = 18.2); 94.3% were carried out within 5 years of diagnosis whilst 5.7% received an intervention after 5 years, latest being performed 88 months following diagnosis. Differences in baseline characteristics amongst patient groups are outlined in Online Resource 5. Forest plots can be found in Online Resource 7.

##### Baseline radiological characteristics, growth dynamics and symptom development

IPD were available for 137 patients, 89 (8.6%) of whom had known symptom status by the end of follow-up; mean duration was 39.7 months (SD = 27.7). 17 developed symptoms while 72 remained asymptomatic. Average follow-up time did not differ between the two groups (41.6 vs. 39.2 months, p = 0.753). Differences in radiological characteristics and growth dynamics are shown in Table 4.

**Table 4:** Growth dynamics and symptom development during active monitoring stratified by baseline characteristics

Factor		Mean AGR (cm^3^/year)	P	Mean RGR (%/year)	P	Symptom development, yes/total (%)	OR (95% CI)	MLR P
Location	Non-skull base	2.14	0.942	53.8	0.213	12/64 (18.8)		0.927
	Skull base	1.79		30.5		5/25 (20.0)		
Diameter	≥ 3.0 cm	4.00	< 0.001	28.4	0.863	15/27 (56.6)	34.90 (5.17–160.40)	0.001
	< 3.0 cm	0.62		27.3		2/62 (3.2)		
Calcification	No	2.42	0.499	38.0	0.093	10/47 (21.3)		0.879
	Yes	1.35		60.6		6/25 (24.0)		
Tumor signal intensity	Hyperintense	2.04	0.988	53.0	0.262	11/41 (26.8)		0.866
	Iso/hypointense	2.02		36.1		4/27 (14.8)		
Peritumoral edema	Yes	0.34	0.301	55.4	0.727	5/10 (50.0)	8.72 (0.35–14.90)	0.027
	No	2.32		44.7		12/63 (19.0)		

*AGR* annual growth rate, *RGR* relative growth rate, *MLR* multi-level regression

#### Surgery

Overall, 741/2050 (36.1%) patients had surgery; 560 were operated at initial presentation whilst 181 were monitored prior to intervention.

##### Extent of resection

Extent of resection was reported in 300/741 (40.5%) patients. GTR was achieved in 285 (95.0%) whereas STR was performed in 10 (5.0%).

##### Post-operative morbidity and mortality

Of 533 (71.9%) patients with available post-operative morbidity data, 88 had complications (pooled proportion = 11.8% [95% CI 3.7–23.5], I^2^ = 91.7%), which were neurological in 47 (53.4%), surgical in 28 (31.8%) and medical in 13 (14.8%) patients. Mortality data was not reported.

##### Histopathology and recurrence

Histology reports were available for 316 patients; 303 had WHO grade I meningioma (pooled proportion = 94.0% [95% CI 88.2–97.9], I^2^ = 54.6%), 10 (3.16%) had WHO grade II meningioma whilst in 3 (0.95%) the pathology revealed WHO grade III meningioma. There was no tumor recurrence observed in 105 patients during a weighted mean follow-up time of 20 months (SD = 14.2) (pooled proportion = 0.3% [95% CI 0.2–2.2], I^2^ = 0.0%).

#### Stereotactic radiosurgery

SRS was the initial treatment in 450 patients. Thirty patients were subject to active monitoring and subsequently underwent SRS due to clinical or radiological progression.

##### Treatment parameters

Two studies provided data regarding radiosurgical parameters. In one study, the treated mean tumor volume was 3.0 cm^3^ (range 0.2–10.4), and the median marginal dose was 14.5 Gray (Gy) (range 12–20) [23]. In the other, the treated median tumor volume was 2.7 cm^3^ (range 0.2–10.5). The median prescription dose was 13 Gy (range 10–18 Gy) with a median prescription isodose line of 50% (range 40–60%). The median tumor coverage was 98% (range 81–100%) and the median values (range) of the homogeneity, conformity and gradient indices were 2.0 (1.7–2.5), 1.5 (1.1–2.2), and 2.8 (2.4–3.7), respectively [25]. No other treatment parameters were reported.

##### Post-SRS complications

Of 389 patients with available data, 85 patients had the following complications (pooled proportion = 32.0% [95% CI 10.6–70.5], I^2^ = 96.3%): headache (n = 22), epilepsy (n = 11), motor deficit (n = 6), scalp paresthesia (n = 6), alopecia (n = 5), dizziness (n = 5), ocular pain (n = 4) and tinnitus (n = 4). Twenty-two patients suffered from asymptomatic peritumoral edema.

##### Response rates

Radiological response was available for 389/480 (81.0%) patients. 382 (98.2%) meningiomas remained stable whilst 7 demonstrated progression during a weighted mean follow-up time of 40.9 months (SD = 14.6) (pooled proportion = 1.5% [95% 0.1–4.3], I^2^ = 96.3%). Adjuvant SRS was performed in two surgery patients for whom progression was not reported.

### Meningioma specific mortality

Two patients exhibited significant meningioma growth at 12 and 88 months of diagnosis and were subsequently offered intervention but declined. The first patient consequently suffered large intracerebral hemorrhage and died. The post-mortem revealed an angioblastic meningioma, more recently defined as hemangiopericytoma. Further details surrounding the 2nd patient’s death were not available.

### QoL and NCF outcomes

One study examined NCF and QoL in 21 patients (mean age 63.4 years) and compared them to a matched healthy population [30]. Meningioma patients fared worse compared to healthy controls on working memory and motor speed. However, there was no correlation between these findings and baseline characteristics. The investigators used the Short Form (36) health survey (SF-36) to assess QoL. The meningioma cohort had lower scores on 2/8 domains namely vitality and general health. A retrospective study of 48 patients (median age 80 years), utilizing a different battery of NCF measures, found no differences in outcomes compared to a matched population [31]. However, when stratified by tumor location, patients with infratentorial meningiomas performed worse on memory and verbal fluency tests. Patients in both studies were treatment-naive. No studies examined the effect of surgery or SRS on NCF and QoL in incidental meningioma.

### Quality and bias assessment results

Quality assessment results for each individual study are summarized in Online Resource 8. Ten studies were rated “good”, eight were “fair” and two were “poor”. As per the GRADE framework, the quality of evidence informing each pooled risk was deemed to be very low (n = 6) or low (n = 1) (Table 5). The generated funnel plots were suspicious for potential reporting bias when pooling the estimate proportions of intervention (treatment arm: active monitoring) and WHO grade (treatment arm: surgery), however, Harbord’s and Begg’s tests (p > 0.05) did not indicate statistically significant bias (Online Resource 9). Reporting bias for studies informing SRS outcomes could not be performed due to the low number of studies (n = 2).

**Table 5:** Level of evidence informing each primary outcome assessed using the GRADE framework

Management	Outcome	Pooled risk (95% CI)^a^	No. of studies (no. of patients)	Quality assessment						
				Type of evidence	Risk of bias	Heterogeneity	Directness	Precision	Reporting bias	Overall
Active monitoring	Symptom development	8.1% (2.7–16.1)	12 (608)	+ 2	− 1	− 1	0	0	0	⨁**◯◯◯** Very low
	Intervention	24.8% (7.5–48.0)	13 (971)	+ 2	− 1	− 1	0	− 1	0	⨁**◯◯◯** Very low
Surgery	Morbidity	11.8% (3.7 23.5)	5 (533)	+ 2	− 1	− 1	0	0	0	⨁**◯◯◯** Very low
	WHO grade I	94.0% (88.2–97.9)	9 (316)	+ 2	− 1	0	− 1^b^	0	0	⨁**◯◯◯** Very low
	Recurrence	0.3% (0.2–2.2)	2 (105)	+ 2	− 1	+ 1	0	0	0	⨁⨁**◯◯** Low
SRS	Morbidity	32.0% (10.6–70.5)	2 (389)	+ 2	0	− 1	0	− 1	NA^c^	⨁**◯◯◯** Very low
	Recurrence	1.5% (0.1–4.3)	2 (389)	+ 2	0	− 1	0	0	NA^c^	⨁**◯◯◯** Very low

The overall quality score was determined based on the sum of the included domains. Type of evidence was based on design of the included studies (+ 2 or + 4); + 2 equates to observational cohort studies and + 4 to randomized controlled trials. Risk of bias score reflected the selection process, measures and outcomes definitions and general methodological and statistical concerns across studies informing each outcome (range − 2 to 0). Heterogeneity was scored using the corresponding I^2^ statistic; low (≤ 25%) = + 1, moderate (~ 50%) = 0 and high (≥ 75%) = − 1. Directness was graded using PICO taking into consideration year of publication and the use of surrogate outcomes (range − 2 to 0). Precision was based on width of the 95% CI. Reporting bias was categorised into detected (+ 1) and not detected (− 1) and performed for each treatment arm using the outcome with the greatest number of studies. The overall quality for each outcome was considered high (≥ 4 points), moderate (3 points), low (2 points) or very low (≤ 1 point)

*NA* non-assessable, *SRS* stereotactic radiosurgery, *WHO* World Health Organization

^a^Obtained by random effects model

^b^Regrading of meningiomas according to the 2016 WHO classification system of brain tumours could not be performed

^c^Could not be assessed due to the low number of studies

## Discussion

This systematic review was conducted to evaluate the current management strategies for incidental meningiomas. Twenty studies, comprising 2130 patients, were included. At initial presentation, half were actively monitored, 27% underwent surgery and 22% were treated with SRS.

### Clinical implications

Active monitoring of incidental meningioma is becoming increasingly common [32], with recent guidelines advocating its use in asymptomatic patients demonstrating no radiological mass effect [3]. However, while the guidelines recommend annual monitoring after a 6-month scan, they do not provide advice regarding the length of follow-up. This stems from the marked heterogeneity of active monitoring regimens, compounded by poor reporting of time to meningioma progression. Moreover, while the development of symptoms is a clear indication for treatment, no consensus exists as to what constitutes significant meningioma growth and when it may necessitate intervention. To this end, we analaysed growth dynamics for meningiomas greater or less than 3 cm. No significant difference was present in the relative growth rate; however, meningiomas ≥ 3 cm demonstrated a higher absolute growth rate (AGR) and a greater risk of clinical progression. This may suggest the use of AGR as a more clinically relevant measure of incidental meningioma growth, which has also been suggested to correlate with a higher WHO meningioma grade [33].

Only a quarter of patients undergoing active monitoring demonstrate radiological or clinical progression requiring intervention. Since most incidental meningiomas are histologically benign and the risk of treatment-related morbidity from surgery or radiosurgery is similar to those reported for symptomatic meningiomas [34–37], a policy of active monitoring seems justified. Meningioma progression and subsequent treatment intervention in the vast majority of cases occurs within 5 years of diagnosis. Given the current lack of consensus on the required duration of monitoring, this suggests that for those patients who do not have radiological and/or clinical progression, imaging surveillance beyond 5-years may be less frequently required or unnecessary depending on individual patient demographics including comorbidity. However, it is important to consider these results within the context of meningioma location. Skull base meningioma constitutes a different clinical and biologic disease entity [38, 39], and the absence of a significant prognostic effect is most likely due to skull-base tumors comprising a minority of truly asymptomatic meningiomas.

Patients with ≥ 3 cm meningiomas and those with peritumoral edema are at a higher risk of symptom development and should therefore be more frequently monitored within the first 5 years of diagnosis. However, an early scan at 3 months is not necessary to rule out metastatic disease as no tumors were reported to be metastatic lesions radiologically misdiagnosed as a meningioma [40]. An argument could also be made for early intervention in these cases; however, the optimal timing remains unclear and we envisage a cost-effectiveness analysis comparing early and delayed (on evidence of progression) treatment would help inform this decision.

### Comparisons to published systematic reviews

One review assessed the risk factors for development of new or worsened symptoms during follow-up of untreated meningiomas, concluding that patients with bigger meningiomas are more likely to become symptomatic; a finding which we replicated [41]. Slow meningioma growth was associated with low T2 signal and non-calcification in previous reviews, which attempted a meta-analysis of the presence or absence of meningioma growth during follow-up [42]. Our analysis demonstrated that high T2 signal and non-calcification were associated with symptom development and intervention respectively, which may have been due to meningioma growth.

### Limitations

Studies included were retrospective of poor quality and this was evident in the highly heterogeneous meta-analyses and the overall very low level of evidence contributing to each primary outcome. Those studies also spanned a 27-year time-period during which major advances in neurosurgical technology and management guidelines occurred, introducing confounding variables into our analysis. Duration of follow-up for patients subject to active monitoring was not available in all studies and the weighted duration of ~ 5 years indicates that long-term prognosis of incidental meningioma remains unclear. Morbidity could not be stratified based on intervention time—immediate and delayed, which prevented a detailed analysis of post-treatment outcomes. Moreover, severity could not be graded and thus complications were grouped together preventing appropriate estimation of the risk of serious morbidity. This is important since the pooled morbidity risk following SRS was three times the risk following surgery. This is likely due to inclusion of symptoms such as headache, which are seldom reported as side effects in the surgical meningioma literature. These were however maintained in our results as for asymptomatic patients undergoing treatment, a headache which was not present pre-intervention, and which might affect QoL, could be regarded as unfavourable outcome. Moreover, it was not among our objectives to compare and contrast the morbidity of surgery and SRS with the purpose of determining the best intervention for incidental meningioma, but rather to demonstrate that complications do occur and considering most incidental meningiomas remain stable during follow-up, this would disincentivize intervention at initial diagnosis.

### Future research

Our analysis shows that there are a wide variety of measurements of meningioma growth. A uniform definition is therefore required for ongoing clinical management and future research. Furthermore, the current heterogeneity in the frequency of radiological surveillance requires a unified strategy in order to better identify radiological progression and reduce the costs to healthcare. All studies in this review were retrospective in nature and single-institution. There is a clear need to define a validated core outcome set for data standardization and its subsequent use in prospective multi-center studies investigating the management and outcomes of incidental meningiomas.

## Conclusions

Incidental, asymptomatic intracranial meningiomas are common with no clear consensus on the optimal management strategy. This review demonstrates a wide variation in current clinical practice. Considering that most incidental meningiomas are WHO grade I and reported treatment-related morbidity, the decision to intervene at initial presentation should not be made lightly. Active monitoring might constitute a better strategy and this study shows that the majority of patients who develop clinical or radiological progression will do so within the first 5 years of diagnosis. Regular monitoring may therefore be less frequently required beyond this time point. However, the data used to reach this conclusion has methodological shortcomings. Large prospective multi-center studies are required to develop management algorithms that minimize unnecessary treatment and optimize patient outcomes and quality of life.

## Electronic supplementary material

Below is the link to the electronic supplementary material.

Online Resource 1 (DOCX 17 KB)

Online Resource 2 (DOCX 52 KB)

Online Resource 3 (DOCX 17 KB)

Online Resource 4 (DOCX 46 KB)

Online Resource 5 (DOCX 18 KB)

Online Resource 6 (DOCX 232 KB)

Online Resource 7 (DOCX 218 KB)

Online Resource 8 (DOCX 24 KB)

Online Resource 9 (DOCX 40 KB)

## References

[1] Morris Z, Whiteley WN, Longstreth WT, Weber F, Lee YC, Tsushima Y, Alphs H, Ladd SC, Warlow C, Wardlaw JM, Al-Shahi Salman R (2009). Incidental findings on brain magnetic resonance imaging: systematic review and meta-analysis. BMJ.

[2] Hayward R (2003). VOMIT (victims of modern imaging technology)—an acronym for our times. BMJ.

[3] Goldbrunner R, Minniti G, Preusser M, Jenkinson MD, Sallabanda K, Houdart E, von Deimling A, Stavrinou P, Lefranc F, Lund-Johansen M, Moyal EC, Brandsma D, Henriksson R, Soffietti R, Weller M (2016). EANO guidelines for the diagnosis and treatment of meningiomas. Lancet Oncol.

[4] de Boer AW, Drewes YM, de Mutsert R, Numans ME, den Heijer M, Dekkers OM, de Roos A, Lamb HJ, Blom JW, Reis R (2017). Incidental findings in research: A focus group study about the perspective of the research participant. J Magn Reson Imaging.

[5] Liberati A, Altman DG, Tetzlaff J, Mulrow C, Gøtzsche PC, Ioannidis JPA, Clarke M, Devereaux PJ, Kleijnen J, Moher D (2009). The PRISMA statement for reporting systematic reviews and meta-analyses of studies that evaluate healthcare interventions: explanation and elaboration. BMJ.

[6] Fountain DM, Soon WC, Matys T, Guilfoyle MR, Kirollos R, Santarius T (2017). Volumetric growth rates of meningioma and its correlation with histological diagnosis and clinical outcome: a systematic review. Acta Neurochir (Wien).

[7] Barendregt JJ, Doi SA, Lee YY, Norman RE, Vos T (2013). Meta-analysis of prevalence. J Epidemiol Community Health.

[8] National Institute of Health National Heart, Lung and Blood Institute Quality Assessment Tool for Observational Cohort and Cross-Sectional Studies. https://www.nhlbi.nih.gov/health-pro/guidelines/in-develop/cardiovascular-risk-reduction/tools/cohort. Accessed 11 April 2011

[9] Begg CB, Mazumdar M (1994). Operating characteristics of a rank correlation test for publication bias. Biometrics.

[10] Harbord RM, Egger M, Sterne JA (2006). A modified test for small-study effects in meta-analyses of controlled trials with binary endpoints. Stat Med.

[11] Duval S, Tweedie R (2000). Trim and fill: a simple funnel-plot-based method of testing and adjusting for publication bias in meta-analysis. Biometrics.

[12] Firsching RP, Fischer A, Peters R, Thun F, Klug N (1990). Growth rate of incidental meningiomas. J Neurosurg.

[13] Olivero WC, Lister JR, Elwood PW (1995). The natural history and growth rate of asymptomatic meningiomas: a review of 60 patients. J Neurosurg.

[14] Go RS, Taylor BV, Kimmel DW (1998). The natural history of asymptomatic meningiomas in Olmsted County, Minnesota. Neurology.

[15] Nishizaki T, Ozaki S, Kwak T, Ito H (1999). Clinical features and surgical outcome in patients with asymptomatic meningiomas. Br J Neurosurg.

[16] Niiro M, Yatsushiro K, Nakamura K, Kawahara Y, Kuratsu JI (2000). Natural history of elderly patients with asymptomatic meningiomas. J Neurol Neurosurg Psychiatry.

[17] Yoneoka Y, Fujii Y, Tanaka R (2000). Growth of incidental meningiomas. Acta Neurochir.

[18] Nakamura M, Roser F, Michel J, Jacobs C, Samii M, De Tribolet N, George B, Brem H, Weaver K, Kaye AH (2003). The natural history of incidental meningiomas. Neurosurgery.

[19] Sonoda Y, Sakurada K, Saino M, Kondo R, Sato S, Kayama T (2005). Multimodal strategy for managing meningiomas in the elderly. Acta Neurochirurgica.

[20] Reinert M, Babey M, Curschmann J, Vajtai I, Seiler RW, Mariani L (2006). Morbidity in 201 patients with small sized meningioma treated by microsurgery. Acta neurochirurgica.

[21] Yano S, Kuratsu JI (2006). Indications for surgery in patients with asymptomatic meningiomas based on an extensive experience. J Neurosurg.

[22] Hashiba T, Hashimoto N, Izumoto S, Suzuki T, Kagawa N, Maruno M, Kato A, Yoshimine T (2009). Serial volumetric assessment of the natural history and growth pattern of incidentally discovered meningiomas. J Neurosurg.

[23] Jo KW, Kim CH, Kong DS, Seol HJ, Nam DH, Park K, Kim JH, Lee JI (2011). Treatment modalities and outcomes for asymptomatic meningiomas. Acta Neurochir.

[24] Kasuya H, Kubo O, Kato K, Krischek B (2012). Histological characteristics of incidentally-found growing meningiomas. J Med Investig.

[25] Hoe Y, Choi YJ, Kim JH, Kwon DH, Kim CJ, Cho YH (2015). Peritumoral brain edema after stereotactic radiosurgery for asymptomatic intracranial meningiomas: risks and pattern of evolution. J Korean Neurosurg Soc.

[26] Jadid KD, Feychting M, Hoijer J, Hylin S, Kihlstrom L, Mathiesen T (2015). Long-term follow-up of incidentally discovered meningiomas. Acta Neurochir.

[27] Liu Y, Li F, Wang C (2015). Clinical features and surgical treatment of asymptomatic meningiomas. Turk Neurosurg.

[28] Zeng L, Wang L, Ye F, Chen J, Lei T, Chen J (2015). Clinical characteristics of patients with asymptomatic intracranial meningiomas and results of their surgical management. Neurosurg Rev.

[29] Rubin G, Herscovici Z, Laviv Y, Jackson S, Rappaport ZH (2011). Outcome of untreated meningiomas. Isr Med Assoc J.

[30] Van Nieuwenhuizen D, Ambachtsheer N, Heimans JJ, Reijneveld JC, Peerdeman SM, Klein M (2013). Neurocognitive functioning and health-related quality of life in patients with radiologically suspected meningiomas. J Neuro-oncol.

[31] Butts AM, Weigand S, Brown PD, Petersen RC, Jack CR, Machulda MM, Cerhan JH (2017). Neurocognition in individuals with incidentally-identified meningioma. J Neuro-oncol.

[32] Agarwal V, McCutcheon BA, Hughes JD, Carlson ML, Glasgow AE, Habermann EB, Nguyen QB, Link MJ, Van Gompel JJ (2017). Trends in management of intracranial meningiomas: analysis of 49,921 cases from modern cohort. World Neurosurg.

[33] Soon WC, Fountain DM, Koczyk K, Abdulla M, Giri S, Allinson K, Matys T, Guilfoyle MR, Kirollos RW, Santarius T (2017). Correlation of volumetric growth and histological grade in 50 meningiomas. Acta Neurochir (Wien).

[34] Chang JH, Chang JW, Choi JY, Park YG, Chung SS (2003). Complications after gamma knife radiosurgery for benign meningiomas. J Neurol Neurosurg Psychiatry.

[35] Boviatsis EJ, Bouras TI, Kouyialis AT, Themistocleous MS, Sakas DE (2007). Impact of age on complications and outcome in meningioma surgery. Surg Neurol.

[36] Kuhn EN, Taksler GB, Dayton O, Loganathan A, Bourland D, Tatter SB, Laxton AW, Chan MD (2014). Is there a tumor volume threshold for postradiosurgical symptoms? A single-institution analysis. Neurosurgery.

[37] Sicking J, Voss KM, Spille DC, Schipmann S, Holling M, Paulus W, Hess K, Steinbicker AU, Stummer W, Grauer O, Wolfer J, Brokinkel B (2018). The evolution of cranial meningioma surgery-a single-center 25-year experience. Acta Neurochir (Wien).

[38] Meling TR, Da Broi M, Scheie D, Helseth E (2018). Meningiomas: skull base versus non-skull base. Neurosurg Rev.

[39] Clark VE, Erson-Omay EZ, Serin A, Yin J, Cotney J, Ozduman K, Avsar T, Li J, Murray PB, Henegariu O, Yilmaz S, Gunel JM, Carrion-Grant G, Yilmaz B, Grady C, Tanrikulu B, Bakircioglu M, Kaymakcalan H, Caglayan AO, Sencar L, Ceyhun E, Atik AF, Bayri Y, Bai H, Kolb LE, Hebert RM, Omay SB, Mishra-Gorur K, Choi M, Overton JD, Holland EC, Mane S, State MW, Bilguvar K, Baehring JM, Gutin PH, Piepmeier JM, Vortmeyer A, Brennan CW, Pamir MN, Kilic T, Lifton RP, Noonan JP, Yasuno K, Gunel M (2013). Genomic analysis of non-NF2 meningiomas reveals mutations in TRAF7, KLF4, AKT1, and SMO. Science.

[40] National Comprehensive Cancer Network (2017) NCCN clinical practice guidelines in oncology-central nervous system cancer (version 1.2017). https://www.nccn.org/professionals/physician_gls/pdf/cns.pdf. Accessed 11 Nov 2017

[41] Sughrue ME, Rutkowski MJ, Aranda D, Barani IJ, McDermott MW, Parsa AT (2010). Treatment decision making based on the published natural history and growth rate of small meningiomas. J Neurosurg.

[42] Zeng L, Liang P, Jiao J, Chen J, Lei T (2015). Will an asymptomatic meningioma grow or not grow? A meta-analysis. J Neurol Surg A.

