# Correction to: Incidental intracranial meningiomas: a systematic review and meta-analysis of prognostic factors and outcomes

**DOI:** 10.1007/s11060-019-03237-5

**Published:** 2019-07-31

**Authors:** Abdurrahman I. Islim, Midhun Mohan, Richard D. C. Moon, Nisaharan Srikandarajah, Samantha J. Mills, Andrew R. Brodbelt, Michael D. Jenkinson

**Affiliations:** 1 Institute of Translational Medicine, University of Liverpool, Liverpool, UK; 2 Faculty of Health and Life Sciences, University of Liverpool, Liverpool, UK; 3 Department of Neurosurgery, The Walton Centre NHS Foundation Trust, Liverpool, UK; 4 Department of Neuroradiology, The Walton Centre NHS Foundation Trust, Liverpool, UK

## Correction to: Journal of Neuro-Oncology (2019) 142:211–221 10.1007/s11060-019-03104-3

Issues with data analysis have recently been highlighted by a reader of our article. These have been addressed with changes to Tables 2 and 4, as shown below, and Online Resources 5–7. T2 and peritumoral signal are no longer prognostic factors on simple pooled (Online Resource 5) and IPD (Table 4) analyses respectively. In Table 5, the number of patients which informed the outcomes symptom development and intervention were 575 and 947 respectively; 69 developed symptoms (pooled proportion %8.4 [95% CI 2.8–16.7], I^2^ = 88.9%). These included motor and cognitive deficits (n = 1). We apologise to the readership of the Journal of Neuro-Oncology for these errors and thank the reader for helping us identify them.

**Table 2:** Baseline clinical and radiological characteristics

No. of studies informing characteristic	No. of valid cases informing characteristic (%)	Characteristics		Total	Surgery	SRS	Active monitoring	P
18	2050	N. of patients (%)		2050	560 (27.3)	450 (22.0)	1040 (50.7)	
13	803 (39.2)	Mean age, years (SD)		63.1 (6.9)	61.5 (4.7)	54.9 (NR)^b^	64 (6.9)	< 0.001
17	1948 (95.0)	Sex, N (%)	Female	1549	369 (23.8)	363 (23.4)	817 (52.7)	0.539
			Male	399	89 (22.3)	87 (21.8)	223 (55.9)	
16	1495 (72.9)	Location, N (%)^a^	Non-skull base	1024	277 (27.1)	235 (22.9)	512 (50.0)	< 0.001
			Convexity	431	126	86	219	
			Parafalcine	245	61	71	113	
			Parasagittal	151	42	36	73	
			Tentorial	62	12	28	22	
			Intraventricular	26	4	12	10	
			Skull base	471	106 (22.5)	154 (32.7)	211 (44.8)	
			Anterior midline	112	22	43	47	
			Sphenoid wing	98	25	11	62	
			Posterior fossa - lateral and posterior	52	23	12	17	
			Posterior fossa - midline	138	17	87	34	
15	888 (43.3)	Mean diameter, cm (SD)		2.14 (0.61)	2.11 (0.42)	1.73 (NR)^b^	2.19 (0.66)	<0.001
10	615 (30.0)	Calcification, N (%)	No	394	64 (16.2)	NR	330 (83.8)	0.177
			Yes	221	27 (12.2)	NR	194 (87.8)	
5	298 (14.5)	Tumor signal intensity, N (%)	Hyperintense	120	40 (33.3)	NR	80 (66.6)	0.237
			Iso/hypointense	178	48 (27.0)	NR	130 (73.0)	
12	1107 (54.0)	Peritumoral edema, N (%)	Yes	144	47 (32.6)	24 (16.7)	73 (50.7)	
			No	963	145 (15.1)	365 (37.9)	453 (47.0)	

*NR* not reported, *SRS* stereotactic radiosurgery

^a^One study which dichotomized location into supratentorial and infratentorial was excluded [15]

^b^Available in one study which did not report SD [25]

**Table 4:** Growth dynamics and symptom development during active monitoring stratified by baseline characteristics

Factor		Mean/median AGR (cm^3^/year)	P	Mean/median RGR (%/year)	P	Symptom development, yes/total (%)	OR (95% CI)	MLR P^a^
Location	Non-skull base	7.97/0.68	0.747	70.2/17.0	0.405	12/60 (20.0)		0.116
	Skull base	5.82/2.93		28.1/17.2		5/25 (20.0)		
Diameter	≥ 3.0 cm	19.7/5.91	0.004	114/24.7	0.091	15/27 (55.5)	12.16 (5.56–18.78)	< 0.001
	< 3.0 cm	1.56/0.46		31.4/12.7		2/58 (3.45)		
Calcification	No	19.0/19.0	NA	141/141	NA	1/1 (100)		
	Yes	NR		NR		NR		
Tumor signal intensity	Hyperintense	NR	NA	NR	NA	NR		
	Iso/hypointense	NR		NR		NR		
Peritumoral edema	Yes	NR	NA	NR	NA	NR		
	No	2.32/0.62		19.7/13.2		1/29 (3.45)		

*AGR* absolute growth rate, *RGR* relative growth rate, *MLR* multi-level regression, *NA* not applicable, *NR* not reported

^a^Performed to 50 iterations

## Electronic supplementary material

Below is the link to the electronic supplementary material.

Online Resources 5, 6, 7 (DOCX 1364 KB)

