# Supplementary material for: Incidental intracranial meningiomas: a systematic review and meta-analysis of prognostic factors and outcomes
Source: J Neurooncol. 2019 Jan 17;142(2):211–21. doi: 10.1007/s11060-019-03104-3 (PMC6449307; doi:10.1007/s11060-019-03104-3)
Supplement: Supplementary file 1 — Online Resource 1 (DOCX 17 KB) [file 11060_2019_3104_MOESM1_ESM.docx]

Online Resource 1. Search strategy for Medline (Ovid) and Embase (Ovid)

| **Search** | **Query** |
| --- | --- |
| 1 | exp meningioma/ |
| 2 | ((central nervous system or CNS or brain* or cerebral* or intracranial or intra-cranial) adj3 (cancer* or tumo?r* or malignan* or neoplas*)).mp. |
| 3 | 1 or 2 |
| 4 | (glioma* or glial* or glioblastoma* or GBM* or astrocytoma* or ependym* or subependym* or neurocytoma* or pineal* or pineo* or chordoma* or hamartoma* or pituitary* or craniopharyngioma* or neuroblastoma* or medulloblastoma* or lymphoma* or metastat*).mp. |
| 5 | ((lung* or breast* or skin* or blood* or h?ematolg* or dermatolog* or renal* or genitourinary*) adj3 (cancer* or tumo?r* or malignan* or neoplas*)).mp. |
| 6 | (leuk?emia* or myeloma* melanoma*).mp. |
| 7 | 4 or 5 or 6 |
| 8 | 3 not 7 |
| 9 | (asymptomatic or incidental or small or untreated).mp. |
| 10 | (surgery or radiotherapy or radiosurg* or observ* or conservative treatment or follow-up or natural history or growth).mp. |
| 11 | 8 and 9 and 10 |
| 12 | Limit 11 to English |

**Incidental Intracranial Meningiomas: A Systematic Review and Meta-Analysis of Prognostic Factors and Outcomes**

**Journal of Neuro-Oncology**

**Authors and affiliations:**

Abdurrahman I. Islim, MPhil ^1,2,3^

Midhun Mohan, MRes ^2,3^

Richard D.C. Moon, MB, BChir ^2,3^

Nisaharan Srikandarajah, MRCS, MBBS ^1,3^

Samantha J. Mills, PhD ^4^

Andrew R. Brodbelt, PhD ^3^

Michael D. Jenkinson, PhD ^1,3^

1. Institute of Translational Medicine, University of Liverpool, Liverpool, UK
2. Faculty of Health and Life Sciences, University of Liverpool, Liverpool, UK
3. Department of Neurosurgery, The Walton Centre NHS Foundation Trust, Liverpool, UK
4. Department of Neuroradiology, The Walton Centre NHS Foundation Trust, Liverpool, UK

**Corresponding author:**

Abdurrahman I Islim

Email: [a.islim@liv.ac.uk](mailto:a.islim@liv.ac.uk)
