# Supplementary material for: Incidental intracranial meningiomas: a systematic review and meta-analysis of prognostic factors and outcomes
Source: J Neurooncol. 2019 Jan 17;142(2):211–21. doi: 10.1007/s11060-019-03104-3 (PMC6449307; doi:10.1007/s11060-019-03104-3)
Supplement: Supplementary file 3 — Online Resource 3 (DOCX 17 KB) [file 11060_2019_3104_MOESM3_ESM.docx]

Online Resource 3. ICOM classification of meningioma location (unpublished material)

| **Main category** | **Subcategories** |  |  |
| --- | --- | --- | --- |
| Convexity | Anterior^a^ | Posterior^a^ |  |
| Parasagittal | Anterior^a^ | Posterior^a^ | Falco-tentorial |
| Parafalcine | Anterior^a^ | Posterior^a^ | Falco-tentorial |
| Sphenoid wing | Lateral | Medial (including ACP) |  |
| Anterior midline | Cribriform plate or olfactory groove^b^ | Planum^c^ | Tuberculum and diaphragma sellae |
| Post fossa - midline | Clival | Petro-clival | Anterior foramen magnum^d^ |
| Post fossa –  Lateral & posterior | Petrous | Squamous occipital | Posterior foramen magnum^d^ |
| Tentorial | Supratentorial | Infratentorial |  |
| Intraventricular |  |  |  |
| Pineal region^e^ |  |  |  |
| ^a^ The main attachment is located anterior or posterior, respectively, to the coronal suture  ^b^ Arising between the crista galli and the fronto-sphenoid suture  ^c^ Arising between the fronto-sphenoid suture and the limbus sphenoidale  ^d^ The main attachment is located anterior or posterior, respectively, to the hypoglossal canal  ^e^ No obvious tentorial attachment | | | |

**Incidental Intracranial Meningiomas: A Systematic Review and Meta-Analysis of Prognostic Factors and Outcomes**

**Journal of Neuro-Oncology**

**Authors and affiliations:**

Abdurrahman I. Islim, MPhil ^1,2,3^

Midhun Mohan, MRes ^2,3^

Richard D.C. Moon, MB, BChir ^2,3^

Nisaharan Srikandarajah, MRCS, MBBS ^1,3^

Samantha J. Mills, PhD ^4^

Andrew R. Brodbelt, PhD ^3^

Michael D. Jenkinson, PhD ^1,3^

1. Institute of Translational Medicine, University of Liverpool, Liverpool, UK
2. Faculty of Health and Life Sciences, University of Liverpool, Liverpool, UK
3. Department of Neurosurgery, The Walton Centre NHS Foundation Trust, Liverpool, UK
4. Department of Neuroradiology, The Walton Centre NHS Foundation Trust, Liverpool, UK

**Corresponding author:**

Abdurrahman I Islim

Email: [a.islim@liv.ac.uk](mailto:a.islim@liv.ac.uk)
