# Supplementary material for: Incidental intracranial meningiomas: a systematic review and meta-analysis of prognostic factors and outcomes
Source: J Neurooncol. 2019 Jan 17;142(2):211–21. doi: 10.1007/s11060-019-03104-3 (PMC6449307; doi:10.1007/s11060-019-03104-3)
Supplement: Supplementary file 4 — Online Resource 4 (DOCX 46 KB) [file 11060_2019_3104_MOESM4_ESM.docx]

Online Resource 4. Summary of the Study characteristics of the 20 included studies

| Study authors | Publication date | Study design | Setting | N. of incidental meningioma patients | Median/mean age (yrs.) | Female | Male | Intervention | | | | | |
| --- | --- | --- | --- | --- | --- | --- | --- | --- | --- | --- | --- | --- | --- |
|  |  |  |  |  |  |  |  | Active monitoring | | Surgery | SRS | *f*RT | Hospital discharge |
|  |  |  |  |  |  |  |  | C | CR |  |  |  |  |
| Firsching et al. [[12](#_ENREF_12)] | 1990 | Retro | Single-center | 15 | 68/64.7 | 12 | 3 | 0 | 15 | 0 | 0 | 0 | 0 |
| Olivero et al. [[13](#_ENREF_13)] | 1995 | Retro | Single-center | 60 | NA/66 | 45 | 15 | 15* | 45 | 0 | 0 | 0 | 0 |
| Go et al. [[14](#_ENREF_14)] | 1998 | Retro | Multi-center | 33 | NA | NA | NA | 6* | 27 | 0 | 0 | 0 | 0 |
| Nishizaki et al. [[15](#_ENREF_15)] | 1999 | Retro | Multi-center | 108 | 64.7/NA | 85 | 23 | 0 | 33* | 75 | 0 | 0 | 0 |
| Niiro et al. [[16](#_ENREF_16)] | 2000 | Retro | Multi-center | 40 | NA/76.1 | 32 | 8 | 0 | 40 | 0 | 0 | 0 | 0 |
| Yoneoka et al. [[17](#_ENREF_17)] | 2000 | Retro | Single-center | 71 | NA/61 | 61 | 10 | 0 | 37* | 23 | 0 | 0 | 11* |
| Nakamura et al. [[18](#_ENREF_18)] | 2003 | Retro | Single-center | 47 | 59/60.1 | 42 | 5 | 0 | 47 | 0 | 0 | 0 | 0 |
| Sonoda et al. [[19](#_ENREF_19)] | 2004 | Retro | Single-center | 16 | 75/74.8 | 7 | 9 | 0 | 11 | 5 | 0 | 0 | 0 |
| Yano et al. [[21](#_ENREF_21)] | 2006 | Retro | Multi-center | 603 | NA | 489 | 114 | 0 | 351 (171) | 191 | 61 | 0 | 0 |
| Reinert et al. [[20](#_ENREF_20)] | 2006 | Retro | Single-center | 102 | NA | NA | NA | 0 | 0 | 102 | 0 | 0 | 0 |
| Hashiba et al. [[22](#_ENREF_22)] | 2009 | Retro | Single-center | 70 | NA/61.6 | 61 | 9 | 0 | 70 | 0 | 0 | 0 | 0 |
| Jo et al. [[23](#_ENREF_23)] | 2010 | Retro | Single-center | 154 | NA/59.2 | 121 | 33 | 0 | 77 | 8 | 69 | 0 | 0 |
| Rubin et al. [[29](#_ENREF_29)] | 2011 | Retro | Single-center | 54 | NA | NA | NA | 0 | 54 | 0 | 0 | 0 | 0 |
| Kasuya et al. [[24](#_ENREF_24)] | 2012 | Retro | Single-center | 69 | NA | 55 | 14 | 0 | 19 | 50 | 0 | 0 | 0 |
| Van Nieuwenhuizen et al. [[30](#_ENREF_30)] | 2013 | CS | Single-center | 21 | NA/63.4 | 17 | 4 | 0 | 21 | 0 | 0 | 0 | 0 |
| Jadid et al. [[26](#_ENREF_26)] | 2014 | Retro | Single-center | 65 | 68/66.6 | 41 | 24 | 0 | 65 | 0 | 0 | 0 | 0 |
| Hoe et al. [[25](#_ENREF_25)] | 2015 | Retro | Single-center | 320 | 56/NA | 260 | 60 | 0 | 0 | 0 | 320 | 0 | 0 |
| Liu et al. [[27](#_ENREF_27)] | 2015 | Retro | Single-center | 122 | NA/58.6 | 83 | 39 | 0 | 104 | 18 | 0 | 0 | 0 |
| Zeng et al. [[28](#_ENREF_28)] | 2015 | Retro | Single-center | 112 | 53/NA | 88 | 24 | 0 | 24* | 88 | 0 | 0 | 0 |
| Butts et al. [[31](#_ENREF_31)] | 2017 | Retro | Multi-center | 48 | 80/NA | 32 | 16 | 0 | 48 | 0 | 0 | 0 | 0 |
| Abbreviations: C=clinical; CR=clinical-radiological; SRS=stereotactic radiosurgery; *f*RT=fractionated radiotherapy; NA=not available; Retro=retrospective; CS=cross-sectional  *not all outcomes were available  () complete outcomes available for a part of this group | | | | | | | | | | | | | |

**Incidental Intracranial Meningiomas: A Systematic Review and Meta-Analysis of Prognostic Factors and Outcomes**

**Journal of Neuro-Oncology**

**Authors and affiliations:**

Abdurrahman I. Islim, MPhil ^1,2,3^

Midhun Mohan, MRes ^2,3^

Richard D.C. Moon, MB, BChir ^2,3^

Nisaharan Srikandarajah, MRCS, MBBS ^1,3^

Samantha J. Mills, PhD ^4^

Andrew R. Brodbelt, PhD ^3^

Michael D. Jenkinson, PhD ^1,3^

1. Institute of Translational Medicine, University of Liverpool, Liverpool, UK
2. Faculty of Health and Life Sciences, University of Liverpool, Liverpool, UK
3. Department of Neurosurgery, The Walton Centre NHS Foundation Trust, Liverpool, UK
4. Department of Neuroradiology, The Walton Centre NHS Foundation Trust, Liverpool, UK

**Corresponding author:**

Abdurrahman I Islim

Email: [a.islim@liv.ac.uk](mailto:a.islim@liv.ac.uk)
