# Supplementary material for: Incidental intracranial meningiomas: a systematic review and meta-analysis of prognostic factors and outcomes
Source: J Neurooncol. 2019 Jan 17;142(2):211–21. doi: 10.1007/s11060-019-03104-3 (PMC6449307; doi:10.1007/s11060-019-03104-3)
Supplement: Supplementary file 8 — Online Resource 8 (DOCX 24 KB) [file 11060_2019_3104_MOESM8_ESM.docx]

## Online Resource 8. Results of the quality assessment performed for all studies included

##

**Incidental Intracranial Meningiomas: A Systematic Review and Meta-Analysis of Prognostic Factors and Outcomes**

**Journal of Neuro-Oncology**

**Authors and affiliations:**

Abdurrahman I. Islim, MPhil ^1,2,3^

Midhun Mohan, MRes ^2,3^

Richard D.C. Moon, MB, BChir ^2,3^

Nisaharan Srikandarajah, MRCS, MBBS ^1,3^

Samantha J. Mills, PhD ^4^

Andrew R. Brodbelt, PhD ^3^

Michael D. Jenkinson, PhD ^1,3^

1. Institute of Translational Medicine, University of Liverpool, Liverpool, UK
2. Faculty of Health and Life Sciences, University of Liverpool, Liverpool, UK
3. Department of Neurosurgery, The Walton Centre NHS Foundation Trust, Liverpool, UK
4. Department of Neuroradiology, The Walton Centre NHS Foundation Trust, Liverpool, UK

**Corresponding author:**

Abdurrahman I Islim

Email: [a.islim@liv.ac.uk](mailto:a.islim@liv.ac.uk)
