# Supplementary material for: Incidental intracranial meningiomas: a systematic review and meta-analysis of prognostic factors and outcomes
Source: J Neurooncol. 2019 Jan 17;142(2):211–21. doi: 10.1007/s11060-019-03104-3 (PMC6449307; doi:10.1007/s11060-019-03104-3)
Supplement: Supplementary file 9 — Online Resource 9 (DOCX 40 KB) [file 11060_2019_3104_MOESM9_ESM.docx]

## Online Resource 9. Reporting bias assessment results


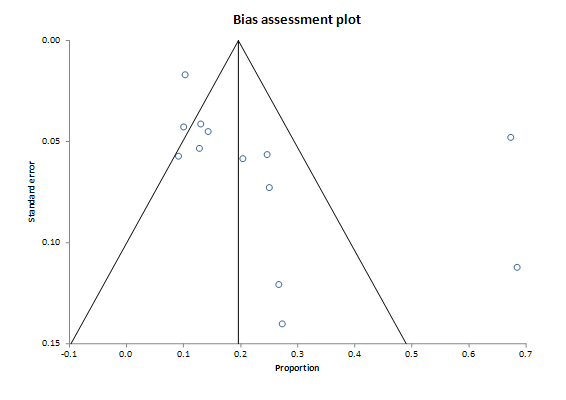


A funnel plot assessing reporting bias for studies contributing to active monitoring outcomes and their reported percentage proportion. Each study is represented by a point and is located at the proportion of intervention against its standard error (SE). Harbord’s test (p=0.710) and Begg’s test (p=0.067) were not statistically significant.

A funnel plot assessing reporting bias for studies contributing to surgery outcomes and their reported percentage proportion. Each study is represented by a point and is located at the proportion of WHO grade I meningioma against its standard error (SE). Harbord’s test (p=0.119) and Begg’s test (p=0.399) were not statistically significant.


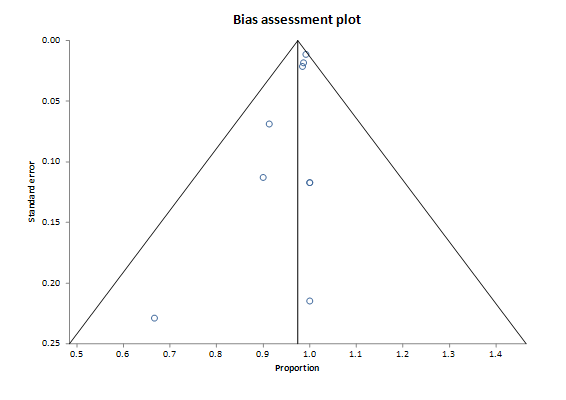


**Incidental Intracranial Meningiomas: A Systematic Review and Meta-Analysis of Prognostic Factors and Outcomes**

**Journal of Neuro-Oncology**

**Authors and affiliations:**

Abdurrahman I. Islim, MPhil ^1,2,3^

Midhun Mohan, MRes ^2,3^

Richard D.C. Moon, MB, BChir ^2,3^

Nisaharan Srikandarajah, MRCS, MBBS ^1,3^

Samantha J. Mills, PhD ^4^

Andrew R. Brodbelt, PhD ^3^

Michael D. Jenkinson, PhD ^1,3^

1. Institute of Translational Medicine, University of Liverpool, Liverpool, UK
2. Faculty of Health and Life Sciences, University of Liverpool, Liverpool, UK
3. Department of Neurosurgery, The Walton Centre NHS Foundation Trust, Liverpool, UK
4. Department of Neuroradiology, The Walton Centre NHS Foundation Trust, Liverpool, UK

**Corresponding author:**

Abdurrahman I Islim

Email: a.islim@liv.ac.uk
