# Supplementary material for: Correction to: Incidental intracranial meningiomas: a systematic review and meta-analysis of prognostic factors and outcomes
Source: J Neurooncol. 2019 Jul 31;144(2):427–9. doi: 10.1007/s11060-019-03237-5 (PMC6700050; doi:10.1007/s11060-019-03237-5)
Supplement: Supplementary file 1 — Online Resources 5, 6, 7 (DOCX 1364 KB) [file 11060_2019_3237_MOESM1_ESM.docx]

Online Resource 5. Differences in baseline characteristics based on symptom development and intervention

| **Table 5. Differences in baseline characteristics based on symptom development and intervention** | | | | | | | | | | | |
| --- | --- | --- | --- | --- | --- | --- | --- | --- | --- | --- | --- |
|  |  | Symptom development | |  | |  | Intervention | |  | | |
| Characteristic |  | Yes | No | Univariate P | RD (95% CI) | I^2^ | Yes | No | Univariate P | RD (95% CI) | I^2^ |
| Age, N (%) | <65 yrs. | 37 (14.6) | 217 (85.4) | 0.028 | -0.03 (-0.11-0.05) | 56% | 133 (40.5) | 195 (59.5) | <0.001 | 0.08 (-0.06-0.21) | 75% |
|  | ≥65 yrs. | 26 (8.6) | 276 (91.4) |  |  |  | 36 (11.4) | 279 (88.6) |  |  |  |
| Sex, N (%) | Male | 24 (17.9) | 110 (82.1) | 0.029 | 0.02 (-0.04-0.07) | 0% | 66 (32.5) | 137 (67.5) | <0.001 | 0.02 (-0.02-0.05) | 1% |
|  | Female | 45 (10.2) | 396 (89.8) |  |  |  | 154 (20.7) | 589 (79.3) |  |  |  |
| Location, N (%) | Non-skull base | 56 (13.3) | 365 (86.7) | 0.154 | 0.01 (-0.03-0.06) | 0% | 150 (31.5) | 326 (68.5) | 0.002 | 0.01 (-0.04-0.06) | 0% |
|  | Skull base | 13 (8.4) | 141 (91.6) |  |  |  | 38 (20.4) | 148 (79.6) |  |  |  |
| Diameter, N (%) | ≥3.0 cm | 24 (36.4) | 42 (63.6) | <0.001 | 0.21 (-0.05-0.47) | 87% | 35 (44.3) | 44 (55.7) | <0.001 | 0.04 (-0.08-0.17) | 57% |
|  | <3.0 cm | 39 (8.0) | 451 (92.0) |  |  |  | 134 (23.8) | 430 (76.2) |  |  |  |
| Calcification, N (%) | No | 47 (15.9) | 248 (84.1) | 0.132 | 0.01 (-0.03-0.06) | 0% | 123 (41.3) | 175 (52.7) | 0.003 | 0.07 (-0.01-0.15) | 62% |
|  | Yes | 21 (11.5) | 161 (88.5) |  |  |  | 53 (28.0) | 136 (72.0) |  |  |  |
| Tumour signal intensity, N (%) | Hyperintense | 9 (13.4) | 57 (86.6) | 0.438 | 0.06 (-0.13-0.25) | 61% | 10 (13.0) | 67 (87.0) | 0.829 | 0.02 (-0.05-0.10) | 0% |
|  | Iso/hypointense | 7 (9.5) | 67 (90.5) |  |  |  | 13 (11.9) | 96 (88.1) |  |  |  |
| Peritumoral edema, N (%) | Yes | 21 (32.3) | 44 (67.7) | <0.001 | 0.11 (-0.10-0.31) | 82% | 26 (40.6) | 38 (59.4) | 0.467 | -0.01 (-0.08-0.05) | 0% |
|  | No | 48 (13.3) | 312 (86.7) |  |  |  | 144 (35.9) | 257 (64.1) |  |  |  |

Online Resource 6. Forest plots of meta-analysis to determine factors associated with symptom development during active monitoring


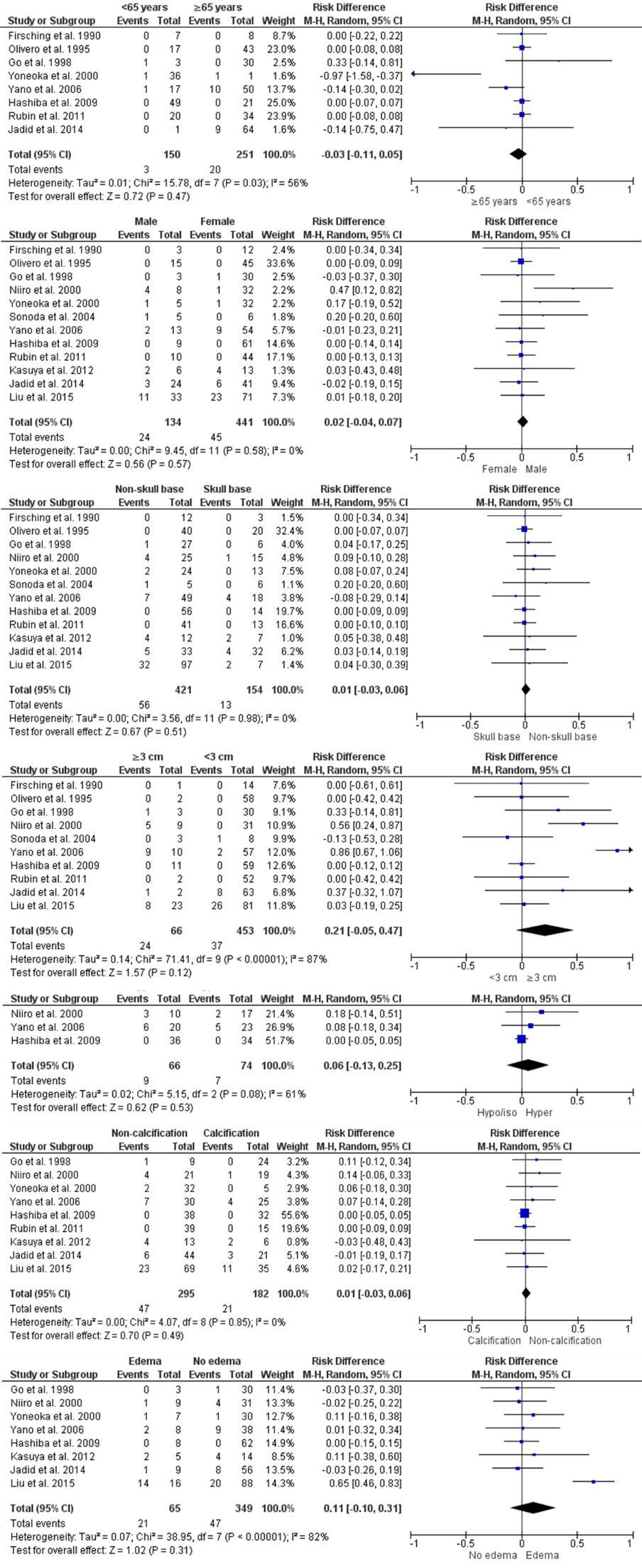


## Online Resource 7. Forest plots of meta-analysis to determine factors associated with intervention after a duration of active monitoring

##
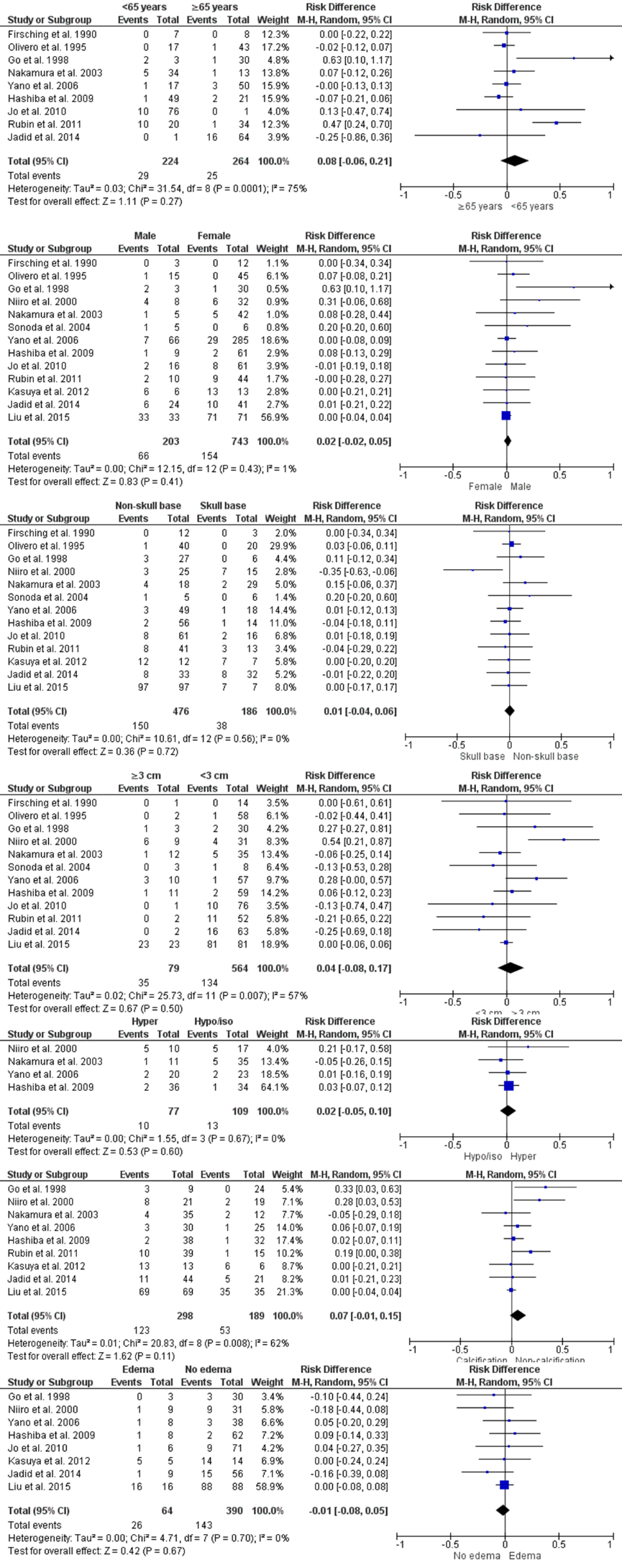


**Incidental Intracranial Meningiomas: A Systematic Review and Meta-Analysis of Prognostic Factors and Outcomes**

**Journal of Neuro-Oncology**

**Authors and affiliations:**

Abdurrahman I. Islim, MPhil ^1,2,3^

Midhun Mohan, MRes ^2,3^

Richard D.C. Moon, MB, BChir ^2,3^

Nisaharan Srikandarajah, MRCS, MBBS ^1,3^

Samantha J. Mills, PhD ^4^

Andrew R. Brodbelt, PhD ^3^

Michael D. Jenkinson, PhD ^1,3^

1. Institute of Translational Medicine, University of Liverpool, Liverpool, UK
2. Faculty of Health and Life Sciences, University of Liverpool, Liverpool, UK
3. Department of Neurosurgery, The Walton Centre NHS Foundation Trust, Liverpool, UK
4. Department of Neuroradiology, The Walton Centre NHS Foundation Trust, Liverpool, UK

**Corresponding author:**

Abdurrahman I Islim

Email: [a.islim@liv.ac.uk](mailto:a.islim@liv.ac.uk)
